# The Risks Associated With Computed Tomography Scans: An Assessment of the Readability and Reliability of Online Text Available for Patient Information and Guidance

**DOI:** 10.7759/cureus.30758

**Published:** 2022-10-27

**Authors:** Monica Hemaya, Maria Hemaya, Amir Habeeb

**Affiliations:** 1 Hospital Medicine, Lister Hospital, East and North Hertfordshire NHS Trust, Stevenage, GBR; 2 Otolaryngology, Cambridge University Hospitals, Cambridge, GBR

**Keywords:** ionising radiation, online medical information, patient information, readability, computed tomography (ct )

## Abstract

Introduction

Computed tomography (CT) scanning has become a fundamental aspect of many diagnostic pathways and therapeutic procedures. However, it is not without risk. Many patients are unaware of the exposure to ionising radiation that is involved with undergoing a CT scan, as well as the associated increase in cancer risk with cumulative exposure. Controversy over which clinician is responsible for advising a patient regarding CT risk often means that patients are left uninformed and unaware. Those who choose to seek further guidance online do so with the risk of encountering poor quality, difficult-to-read medical text, which may leave them even more confused or misinformed.

Aim

This study aimed to assess the readability, quality, and accuracy of the information available to patients online regarding CT scans and their associated risks.

Method

An internet search of 180 websites was collated using three search terms, each entered into three search engines. The terms used were 'CT Risk', 'CT Harm', and 'Dangers of a CT Scan'. Unique websites generated were assessed for readability using four readability formulae: The Flesh-Kincaid Grade Level, the Flesch Reading Ease Score, the Gunning Fog Index, and the Simple Measure of Gobbledygook (SMOG) Index. The text on each website was also evaluated for quality and accuracy using the Discern tool. Mean readability scores were calculated and compared against the defined standard required for the information intended for the general public. A two-tailed t-test was also carried out to assess statistical significance.

Results

Of the 180 websites collated, 77 were unique. 76.62% of websites (59/77) met the readability target for the Flesch-Kincaid Grade Level, and 66.23% (51/77) met the target for the Gunning Fog Index, which was for text to be readable at the Grade 8 level (or by children aged 13-14). 59.74% (46/77) met the target for the SMOG Index, which for healthcare information, was defined as Grade 6 Level, or children aged 11-12. Only 11.69% of websites (9/77) met the target for the Flesch Reading Ease score. 55.84% of websites satisfied the pre-defined standard for three out of four readability criteria, however, only 11.69% satisfied all four criteria, limited by the scores obtained by the Flesch Reading Ease formula. The websites generated a mean Discern score of 3.58, meaning the average quality of the information was deemed to be 'fair', with no serious shortcomings.

Conclusion

More than 50% of websites satisfied three readability criteria simultaneously. However, there is still scope for improvement, both in terms of enhancing the readability of the remaining websites, and also ensuring that all websites review the proportion of polysyllabic words in the text, which is the emphasis of the Flesh Reading Ease Score. In addition, physicians and radiologists have a responsibility to inform patients of the risks associated with CT scans, and to direct them to supplementary good-quality information and resources.

## Introduction

With the advances in medical technology, medical imaging is increasingly being used as a cornerstone for diagnosis. Computed tomography (CT) scans are widely utilised for surgical and medical diagnostics, and even therapeutically for guidance in interventional procedures such as CT-guided biopsies, drainage, and tumour ablation [1]. However, with the growing use of CT scans comes an increase in the associated risks.

CT scans use X-rays to capture thin image slices which are compiled together to create a three-dimensional (3D), cross-sectional view of the intended anatomical structures [2]. The average scan takes between 10 and 20 minutes. To begin, the patient is positioned on a couch which is passed through the 'gantry' of the CT scanner - a ring consisting of an X-ray tube (source of radiation), and radiation detectors directly opposing the X-ray source. The X-ray tube rotates around the patient whilst emitting X-rays, and the detector rotates synchronously so that it is always directly opposite the source. Once emitted, an X-ray photon interacts with the various tissue structures of the body before being received by the detector. Due to the diverse densities of the tissue structures encountered, each tissue responds to the X-ray radiation uniquely. The denser a tissue is, the more X-rays it absorbs, meaning that bone absorbs the most and air the least. This information is received by the detectors and processed by complex computer-based algorithms in order to generate thin image slices. When combined, these produce a 3D cross-sectional image replica of the tissue structures which can then be reviewed and reported by radiologists. Often in practice, there may be a need to enhance particular features of the anatomy such as blood vessels and other soft tissue structures [2]. In these circumstances, a contrast dye may be administered to the patient at the time of the scan.

CT has revolutionised the world of medical imaging. The modality is accessible, widely available, and efficient, with scans being completed in minutes. CT has high spatial resolution and the facility to enhance images with contrast media if required. It has resulted in better treatments and better surgeries [3]. One peer-reviewed report outlined that CT has decreased the need for emergency surgery and has almost made many exploratory surgical procedures extinct, by increasing physicians' level of diagnostic certainty after imaging. Furthermore, CT scanning has reduced the proportion of patients requiring inpatient admission [3]. However, despite the undeniable advantages offered by this imaging modality, it is not risk-free. A single CT scan delivers a much higher dose of radiation to the patient than a plain-film X-ray. For example, the effective radiation dose to the patient from a CT abdomen and pelvis is 10 milliSieverts (mSv), compared to the effective dose from a chest X-ray of 0.02 mSv [4]. Hence, depending on the area being imaged, the radiation doses from CT scans are between 100 to 1,000 times higher than those from conventional X-rays [5].

The main safety concern over CT imaging is the dose of ionising radiation that the patient is exposed to as a result of the scan. X-rays have the potential to cause ionisation of human tissue - that is the ability to convert a neutral atom with no electrical charge, to an ion - an electrically charged unstable entity [6]. This occurs when the X-ray displaces and removes electrons from the atom. Although the ionising power of X-rays is less than that of other types of ionising radiation, X-rays still have low ionising power and hence can cause injury to human cells. If this occurs in the cell's genetic material, this process will lead to mutations in the genetic code. If minimal, the cell may be equipped to repair these. However, cumulative exposure to ionising radiation can result in greater alterations, tissue damage, and the development of more significant diseases such as cancer. One study completed in the United States (US) in 2007 suggested that the radiation from the annual CT scans performed in 2007 would result in 29,000 incident cancers [7]. A further projection made in 2014 suggested that “unless we change our current practices, three percent to five percent of all future cancers may result from exposure to medical imaging” [5]. Most recently, a literature review published in 2021 by The British Institute of Radiology concluded that there is growing evidence from epidemiological data that CT scans can cause cancer, although the precise risk was not conclusively quantified [8]. These concerning figures provoke the question: are all CT scans clinically justifiable? In fact, it is estimated that at least one-quarter of all CT scans might be unnecessary [9], and minimising medically unwarranted CT scans has long been emphasised as one of the key solutions to reducing radiation exposure from medical imaging [10].

Additional concerns surrounding CT imaging include the risks from the administration of intravenous contrast dye used to accentuate certain elements of the anatomy. It is common for critically ill, at-risk patients to suffer from contrast-associated acute kidney injury, which, if not anticipated and mitigated, may cause a deterioration in their clinical state. Occasionally patients will develop allergic reactions following injection or consumption of contrast.

When considering general understanding, one systematic review concluded that patients displayed a lack of awareness and knowledge of the ionising radiation used in medical imaging and had a tendency to underestimate the exposure and associated risk [11]. Furthermore, it highlighted a lack of communication between healthcare professionals and patients regarding radiation exposure and the associated benefits and risks, whilst patients expected to be provided with this information [11]. The global theme discussed in the review was that of informed consent for medical imaging procedures, meaning that patients should be sufficiently advised on what the procedure involves, what the benefits and risks are, what the potential alternatives are, and what would happen if the procedure does not take place. A literature review [12] on such communication found that only 29% of patients in four different studies reported being informed about CT radiation risk or dose. Although it is simple to agree that information should always be provided, there is no consensus on how and by whom this should be done. In one study that explored physicians’ preferences on who should have these discussions [13], 55% of respondents believed that radiologists should provide this communication, whereas 13% felt that this should come from the referring physician. In contrast, more patients expect their referring physicians to discuss these risks with them, rather than radiologists [14]. This research suggests that there is confusion even amongst physicians about how this information would be best delivered. If patients do not feel sufficiently informed by their healthcare providers, it is anticipated that they might seek more information elsewhere.

Due to the wide availability and accessibility of medical information online, it is very often one of the first places that a patient will seek healthcare advice. As demonstrated in a survey done in 2003, 80.3% of UK internet users had used the internet to look up health information [15]. As one of the primary sources of information that is reachable to patients, online medical information has the potential to greatly influence their understanding and decision-making practices. This highlights the need for reliable, readable, and high-quality educational material to be available for patients who choose to advance their medical knowledge online.

Readability is a measure of how easy a piece of text is to read. The level of complexity of the text, its familiarity, legibility, and typography all feed into how readable a given text is [16]. In this study, various objective methods have been employed to assess the readability of online information. Four distinct scores have been used: The Flesch-Kincaid Grade Level, the Flesch Reading Ease Score, the Gunning Fog Index, and The Simple Measure of Gobbledygook (SMOG) Index. Additionally, a further score called the Discern Score has been calculated for each unique website as an indicator of the reliability and accuracy of the information presented.

The Flesch-Kincaid Grade Level and Flesch Reading Ease Score are the most widely tested and used scores for assessing how readable a text is [17]. The Flesch Reading Ease Score gives a text a score between 1 and 100, with 100 being the highest readability score. Scoring between 70 to 80 is equivalent to school grade level 8 in the US, or children aged 13-14. This means the text should be fairly easy for the average adult to read [17]. The Flesch-Kincaid Grade Level assesses the approximate reading grade level of a text, in the context of the US Education System. Text intended for readership by the general public should aim for a grade level of around 8, schooling age 13 to 14.

The Flesch scores are calculated based on two components: sentence length and word length (by number of syllables). Each formula places weight on different components, hence the scales for reporting differ. The formulae for both scores are outlined below [17].

The Flesch Reading Ease Score is calculated by the following equation: 

$$206.835-1.015 \left (\frac{total \, words}{total \, sentences} \right ) - 84.6 \left ( \frac{total \, syllables}{total \, words} \right )$$

The Flesch-Kincaid Grade Level is calculated by the following:

$$0.39 \left ( \frac{total \, words}{total \, sentences} \right ) + 11.8 \left ( \frac{total \, syllables}{total \, words} \right ) - 15.59$$

The Gunning Fog Index generates a score used to measure the clarity and simplicity of the text, by exploring sentence length and complex words. The formula calculates a numerical value between 1 and 20, representing the education level required to understand a text. Similarly to the Flesch-Kincaid Grade Level, the score is based on the US Education System. A Gunning Fog score of 6 is easily readable for sixth-grade students. A text aimed at the public should aim for a grade level of around 8. The text above has a score of 17 has a graduate level [18]. The formula is:

$$0.4 \left [ \left ( \frac{total \, words}{total \, sentences} \right ) + 100 \left ( \frac{complex \, words}{total \, words} \right ) \right ]$$

The SMOG Index is most often used in the healthcare sector. It measures how many years of education the average person needs to have to understand a text, and this is known as the SMOG Grade. Its framework measures complete comprehension of a text [19]. In 2010, a study exploring the readability of online information on Parkinson’s disease found SMOG to be the gold standard readability measure [20]. For healthcare information, the target SMOG Grade should be a reading level of sixth grade or less [21]. The formula to calculate the SMOG Grade is:

$$3 + \sqrt{polysyllabic \, count}$$

The Discern Instrument was specifically created to assess written consumer health information on various treatment choices available from a variety of sources, including the internet. The aim was for it to empower and equip individual patients who were making decisions about their treatment, with a means to ensure the information they were accessing online was accurate and evidence-based [22]. The Discern tool is a 16-item questionnaire that rates the quality of a publication on a scale of 1 to 5. It considers factors such as reliability, relevance, the sources of information used to produce the text, reference to bias or areas of uncertainty, the explanations of each treatment option in question and the benefits and risks of each, any alternative treatment options, and the allowance for shared decision-making. All these elements are reviewed and a final rating from 1 to 5 is assigned, where 1 represents serious or extensive shortcomings, and 5 represents minimal shortcomings.

This study aimed to define the readability and quality of the online information available to patients regarding the risks associated with CT scans.

## Materials and methods

In order to explore the information available online, an internet search was completed over two days in August 2022. Three search phrases were each entered into three search engines: Google, Yahoo, and Microsoft Bing, so there were nine searches made in total. The search phrases entered were ‘CT Risk’, ‘CT Harm’, and ‘Dangers of a CT Scan’. For each search that was made, the top 20 unique results were noted, hence 60 webpages were collated per search term (180 webpages in total). Commercial advertisements were excluded from the search results, as were results irrelevant to the subject of the search term, in order to ensure that only webpages with relevant information were included in the calculations. Information presented in video format was not included in the assessment. Duplicate webpages from the same search were only counted once per search.

Readability scores were calculated for each webpage from each individual search. Following this, mean readability scores were calculated separately for each of the nine searches, then for the unique websites generated by each search term, and finally for the unique websites from all the searches combined. Four well-recognized readability formulae were used as mentioned above: the Flesch-Kincaid Grade Level, the Flesch Reading Ease Score [17], the Gunning Fog Index [18], and the SMOG Index [19-21]. Grades were considered to be at the recommended level x if they were less than or equal to x.9. The full standard used is outlined in Table 1. 

**Table 1:** A summary of the scoring systems and standard used for this study This symbol $\leq$ is used to indicate 'less than or equal to' the number subsequent to it.

Assessment Tool	Target for Readability by the General Public	Standard
Flesch-Kincaid Reading Grade Level	Grade 8	Considered to be at recommended level if $\leq 8.9$
Flesch Reading Ease Score	Score 70-80	70-80
Gunning Fog Index	Grade 8	Considered to be at recommended level if $\leq 8.9$
SMOG Index	Grade 6	Considered to be at recommended level if $\leq 6.9$

The Discern tool was also used to provide a score for the accuracy and reliability of the content on each website. A score was assigned per webpage, and then mean scores were calculated per search, for each group of unique webpages generated by different search terms, and finally for the unique websites from all the searches combined. The final results, therefore, reflect a mean score for each readability measure, for each search, stratified by search term and search engine. A value for standard deviation was also calculated for each mean value, and this was used to generate confidence intervals. Finally, the statistical significance was evaluated using a two-tailed t-test to assess whether differences between search terms as well as search engine readability scores were statistically significant or due to chance.

## Results

Of the 180 results obtained from all the searches, 77 were found to be unique websites. 76.62% of websites (59/77) met the readability target for the Flesch-Kincaid Grade Level, 66.23% (51/77) met the target for the Gunning Fog Index, and 59.74% (46/77) met the target for the SMOG Index. Only 11.69% of websites (9/77) met the target for the Flesch Reading Ease score.

The average readability scores and Discern scores are contained in Table 2, stratified by search term and search engine. Of the total number of unique websites generated (77), the mean Flesch-Kincaid Grade Level was 7.29 (95% confidence interval (CI)=3.76 - 10.81), which is equivalent to the US reading grade level of 7-8, or schooling age 12-14. The average Flesch Reading Ease Score was 56.38 (31.32 - 81.43), which is considered to be fairly difficult to read, or equivalent to 10th-12th grade in the US (15-18 year-olds). The average Gunning Fog Index was 8.51 (4.57 - 12.46), which is at the optimal value for text aimed at the public. The average SMOG Index was 6.84 (4.61 - 9.06), indicating that the average person is required to have an education at the level of US grade 6 to be able to understand the information presented. The average Discern Score was 3.58 (1.20 - 5.97).

**Table 2:** Readability and quality data for three different search terms on popular search engines where 'n' is the number of webpages analysed

Search Term	Search Engine	Flesch–Kincaid Grade Level Mean (95% CI) for n=20	Flesch Reading Ease Score Mean (95% CI) for n=20	Gunning Fog Index Mean (95% CI) for n=20	SMOG Index Mean (95% CI) for n=20	Discern scores Mean (95% CI) for n=20
CT Risk	Google	7.37 (4.44 - 10.29)	55.24 (34.18 - 76.30)	8.61 (4.67 - 12.54)	6.85 (4.70 - 8.99)	4.35 (2.63 - 6.07)
	Yahoo	7.87 (5.37 - 10.36)	52.60 (33.32 - 71.87)	9.25 (6.10 - 12.39)	7.20 (5.14 - 9.26)	3.85 (1.36 - 6.34)
	Bing	8.10 (4.40 - 11.79)	50.55 (25.27 - 75.82)	9.14 (5.12 - 13.16)	7.29 (4.81 - 9.76)	3.85 (1.62 - 6.08)
	Unique Websites n=40	7.59 (4.33 - 10.85)	54.09 (31.55 - 76.62)	8.86 (4.78 - 12.94)	7.04 (4.74 - 9.33)	3.88 (1.60 - 6.15)
CT Harm	Google	7.19 (4.03 - 10.34)	58.29 (37.22 - 79.36)	8.86 (4.91 - 12.81)	7.02 (4.68 - 9.36)	4.45 (2.83 - 6.07)
	Yahoo	7.27 (4.15 - 10.38)	57.21 (33.80 - 80.62)	9.03 (5.61 - 12.44)	6.94 (4.96 - 8.92)	3.40 (0.90 - 5.90)
	Bing	7.17 (3.89 - 10.45)	57.97 (32.41 - 83.52)	9.01 (5.46 - 12.55)	6.78 (4.64 - 8.92)	3.45 (0.95 - 5.95)
	Unique Websites n=37	7.18 (3.99 - 10.37)	57.97 (35.58 - 80.36)	8.83 (5.05 - 12.62)	6.91 (4.68 - 9.13)	3.83 (1.40 - 6.25)
Dangers of a CT Scan	Google	7.33 (3.37 - 11.28)	56.17 (29.55 - 82.79)	8.64 (3.78 - 13.50)	6.90 (4.15 - 9.65)	4.10 (2.10 - 6.10)
	Yahoo	7.43 (4.30 - 10.56)	55.24 (31.97 - 78.51)	8.50 (5.52 - 11.47)	6.89 (5.05 - 8.73)	4.00 (1.71 - 6.29)
	Bing	7.11 (4.40 - 9.81)	57.94 (37.67 - 78.21)	8.15 (5.76 - 10.54)	6.77 (5.02 - 8.51)	4.20 (2.45 - 5.95)
	Unique Websites n=40	7.30 (3.82 - 10.78)	56.62 (31.82 - 81.42)	8.49 (4.60 - 12.38)	6.88 (4.70 - 9.07)	3.92 (1.78 - 6.05)
Total	Total [n=180 websites (unique websites n=77)]	7.29 (3.76 - 10.81)	56.38 (31.32 - 81.43)	8.51 (4.57 - 12.46)	6.84 (4.61 - 9.06)	3.58 (1.20 - 5.97)

For the search specifically relating to the term “CT Risk”, 60 websites were returned from the search via three search engines, and of these 60, 40 were unique websites. The average Flesch-Kincaid Grade Level of these 40 unique websites was 7.59 (4.33 - 10.85), equivalent to school age 12-14. The average Flesch Reading Ease Score was 54.09 (31.55 - 76.62), classed as fairly difficult to read. The average Gunning Fog Index was a grade level of 8.86 (4.78 - 12.94), a level just within the ideal range for text aimed at the public. The SMOG Index average was a Grade Level of 7.04 (4.74 - 9.33), indicating that on average the text from this search was suitably clear for children in 7th Grade (aged 12-13). The average Discern Score was 3.88.

The search for “CT Harm” generated a further 37 unique webpages from the 60 results that were noted. These unique webpages had an average Flesch-Kincaid Grade level of 7.18 (3.99 - 10.37), an average Flesch Reading Ease Score of 57.97 (35.58 - 80.36), and an average Gunning Fog Index of 8.83 (5.05 - 12.62). The mean SMOG Grade was 6.91 (4.68 - 9.13). The mean Discern score was 3.83.

The last search term “Dangers of a CT Scan” generated 40 unique websites from the 60 that were noted. The average Flesch-Kincaid Grade Level was 7.30 (3.82 - 10.78), and the average Flesch Reading Ease Score was 56.62 (31.82 - 81.42). The average Gunning Fog Index was 8.49 (4.60 - 12.38), whilst the SMOG Index average was 6.88 (4.70 - 9.07). The Discern Score for this search term generated an average score of 3.92.

## Discussion

Overall, the websites analysed performed well when assessed against the individually-defined targets for the various readability scores, except for the Flesch Reading Ease Score. The scoring system that generated the highest readability outcome for the online information was the Flesch-Kincaid Grade Level. Of the 77 unique websites, 59 of them (76.62%) met the Flesch-Kincaid Grade Level target of 8. In contrast, the score that estimated the worst readability outcome was the Flesch Reading Ease Score. For each of the three searches that were completed for each search term, the mean Flesch Reading Ease Scores were consistently between 50-60, suggesting they were ‘fairly difficult’ to read, and required the equivalent education of a 10th-12th grade student (15-18 years old) to be understood. Of the unique websites, only nine out of 77 (11.69%) met the target of a Flesch Reading Ease score of 70-80 for readability by the general public.

The mean SMOG grades for each of the three searches for each search term were all between 6.77 - 7.29, equivalent to the reading level of 11-13-year-old school children in the US. However, the target SMOG grade for health information was set by the American Medical Association as being a US Grade 6, in order to be readable by the general public [23]. In this study, the threshold for a target of Grade 6 was any score that was less than or equal to 6.9. Hence, four out of the nine searches generated mean SMOG grades that were beyond this target level. Considering all 77 unique websites, 46 of them (59.74%) met the SMOG Index target of Grade 6 for readability.

The mean Gunning Fog Index of 8.51 suggests the online information met the optimal target of grade 8 (13-14-year-old children) in order to be appropriate for readership by the general public. Of the 77 unique websites, 51 (66.23%) met the Gunning Fog Index target for readability by the general population which, in this study, was any value less than or equal to 8.9. By this measure, the online information regarding CT scanning and its associated risks was deemed to be most readable when assessed by the Flesch-Kincaid Grade level, followed by the Gunning Fog Index. This indicates that the majority of the relevant online information is objectively, by two scoring systems, deemed to be clear and simple to understand. 

Together with the readability scores, a Discern score was assigned to each of the unique websites, as an indicator of the quality and accuracy of the available information. The mean Discern score for all 77 websites was 3.58 (1.20 - 5.97), indicating that on average, the written information was perceived not to have any serious shortcomings, but at worst, only shortcomings that were ‘potentially important’. 

When comparing different search engines, the results demonstrate that Google consistently performed the best on all readability scores for the search term ‘CT Risk”, as well as on the Discern score. However this trend was not consistent for the search terms ‘CT Harm’ and ‘Dangers of a CT Scan’. Bing performed the best on all scores for the term ‘Dangers of a CT Scan’, and for ‘CT Harm’ the highest scores were generated by both Google and Bing searches. The highest mean Discern score of 4.45 was for the Google search of ‘CT Harm’, and the lowest Discern score was for the Yahoo search of ‘CT Harm’. This raises an important and interesting consideration that the patient population may be compromising on the quality and accuracy of the information that they read based on the search engine they use to obtain results.

It is reassuring to discover that Google is performing competitively in terms of the most readable information, however, it is concerning that this trend is not widespread throughout the results. In June 2022, Google had 83.84% of the worldwide desktop market share of search engines, whilst Bing, second in line, had a share of 8.88%, and Yahoo a share of only 2.55% [24]. As the most popular search engine globally, this study identifies growth potential for Google in particular. Better readability and Discern scores for websites collated by Google are likely to have the biggest impact on the patient population. If more patient-targeted information were to be collated by the most used search engine globally, this would likely have a positive impact on patients’ understanding, and would reduce the risk of misinformation from unreliable sources.

This study is deemed to be a reliable and representative assessment of the information available online about CT scans and their associated risks. The search assembled a large sample size of websites, obtained using multiple search terms and search engines, over a defined period. Multiple validated scoring systems were used to measure the readability and quality of the information found. Each scoring system generated results with limited discrepancy between search terms and search engines. There were even similarities between scoring systems.For example, for the search term ‘CT Risk’, the mean Flesch-Kincaid Grade Level score generated for the 40 unique websites was 7.59 (4.33 - 10.85), and the mean SMOG Index for the same websites was comparable at 7.04 (4.74 - 9.33),with overlapping confidence intervals. Although each of these scoring systems had different targets for readability, the fact that they generated similar scores for the same websites increases the reliability and the power of the data obtained. However, caution must be used when comparing results across specific scoring methods due to differences in the scales, such as with the Gunning Fog Index, where the scale ranges from 1 to 20.

Nevertheless despite the strengths of the study, certain limitations are inevitable. With regards to the method, the study design could not possibly account for every search term that may be used by the general public in order to gather information, nor for every search engine. A modified search term via a less recognised search engine may have generated websites that produce very different readability results. Furthermore, this search protocol did not assess websites beyond the first 20 results obtained from each search, which would have taken the author beyond the third page of search results each time. It is possible that there are further websites that satisfy both readability and reliability, but exist after the first 20 searches. The search was not extended to these results as analysis suggests that 75% of search engine users do not click past the first page of results [25] and it was therefore deemed highly unlikely that patients would go beyond the third page of search results. One further limitation to the study was the inability to account for embedded video presentations in the assessment. Some websites displayed excellent video content to complement the written text, however, there was no way to account for this in the study design. Although there may be a wealth of material in such video presentations, the main aim of the study was to assess the written text available, and so whilst it is important to be aware of the undeniable benefit of information available via video presentation, it is fitting that video presentations were not explored in this study. This would however be an interesting avenue for further research in an alternative study. 

Manual removal of websites from the study that were deemed to be irrelevant was done at the author’s discretion, where the subject matter was completely unrelated to CT. For example, a website promoting a Connecticut Drug and Alcohol Awareness Class was removed from the study. Similarly, advertisements were deemed to be biased and financially-driven, and hence were not included in the search results. This may be perceived as both a strength and a weakness to the study. It serves as a strength as irrelevant material was not included in the assessment, and so the opportunity for false skewing of the results was removed. However, it may also be seen as a limitation, as material that is relevant to the general public from their search may be different to what is perceived to be relevant by the study author. The same principle applies when considering that some websites had multiple topics of discussion, such as the risks of positron emission tomography-CT (PET-CT) scans. This information may be confusing to readers who were only after information regarding isolated CT scans, and hence the inclusion of these websites could be controversial.

The scoring systems themselves also have their drawbacks. Although the Discern score is compiled by a thorough 16-item questionnaire, the final score out of 5 is a relatively subjective measure, based on the screening individual’s overall perception of the website’s fulfilment of the Discern tool’s criteria. Due to time constraints, it was only possible for the study to be screened by a single author, which is a further limitation. Associated with this is the risk of human error whilst using the various formulae required to obtain the readability scores.

Despite the limitations of the study, on balance, statistical analysis revealed statistically insignificant differences in the confidence intervals for each readability score when comparisons between search engines and search terms were made (P>0.05). In this study, the consistent overlap of confidence intervals is in fact a benefit rather than a flaw, as it suggests that websites obtained via different search terms and search engines are generating similar legibility results. It further increases the author’s confidence that the true value for readability is 95% likely to lie within the defined intervals. Although the confidence intervals calculated are fairly wide, they were reproducible for each scoring system across the different search terms and search engines. 

Other studies that have explored the readability of clinical information using the same formulae have found that generally, online health information has a readability level that is inappropriate for the use of the general public [26-28]. In contrast, this study has found that the online information about CT and the associated risks was reasonable both in terms of quality and readability, yet there is still significant room for improvement. With the exception of the Flesch Reading Ease Score, 55.84% of websites satisfied the defined standard for the remaining three readability scores outlined in Table 1. When the results for the Flesch Reading Ease Scores are included, only 11.69% of websites satisfied the defined standard for all four readability scores. As the formula for the Flesch Reading Ease Score places more weight on the number of syllables per word, it is possible that the presence of too many polysyllabic words in the online information is the reason for the relatively low Flesch Reading Ease scores. This highlights that a potential area for improvement for authors of medical information is to minimise the use of long, polysyllabic medical terms when writing online for the benefit of patients.

## Conclusions

This study found that approximately half of the websites analysed, 55.84%, satisfied three out of four readability criteria, however, only 11.69% satisfied all four criteria when compared to the pre-defined standard for this study. The quality of information was assessed to be fair, and the average Discern score of 3.58 suggests no serious shortcomings. The average webpage obtained by either of the three search engines was deemed to be readable to the general public as evidenced by the mean readability scores calculated for the Flesch-Kincaid Grade Level, the Gunning Fog Index, and the SMOG Index. Nonetheless, many of these scores are borderline, and confidence intervals straddle the target level in many instances. Improvements are required and provisions should be made to enhance the readability of these webpages even further for members of the public who cannot comprehend the online information, but still have the burden of making the same personal medical decisions.

In addition, it is clear from the research that physicians and radiologists are perceived to have a responsibility to inform patients of the risks associated with CT scans, and to direct them to supplementary good-quality information and resources. The hope is that in the future, this will promote adequately-informed decision-making amongst the patient population.
